# Comparative analysis of autophagy and tauopathy related markers in cerebral ischemia and Alzheimer’s disease animal models

**DOI:** 10.3389/fnagi.2015.00084

**Published:** 2015-05-19

**Authors:** Javier G. Villamil-Ortiz, Gloria P. Cardona-Gomez

**Affiliations:** Cellular and Molecular Neurobiology Area, Group of Neuroscience of Antioquia, Faculty of Medicine, Sede de Investigación Universitaria, University of AntioquiaMedellín, Colombia

**Keywords:** autophagy, tauopathy, Alzheimer’s disease (AD), cerebral ischemia (CI)

## Abstract

Alzheimer’s disease (AD) and cerebral ischemia (CI) are neuropathologies that are characterized by aggregates of tau protein, a hallmark of cognitive disorder and dementia. Protein accumulation can be induced by autophagic failure. Autophagy is a metabolic pathway involved in the homeostatic recycling of cellular components. However, the role of autophagy in those tauopathies remains unclear. In this study, we performed a comparative analysis to identify autophagy related markers in tauopathy generated by AD and CI during short-term, intermediate, and long-term progression using the 3xTg-AD mouse model (aged 6,12, and 18 months) and the global CI 2-VO (2-Vessel Occlusion) rat model (1,15, and 30 days post-ischemia). Our findings confirmed neuronal loss and hyperphosphorylated tau aggregation in the somatosensory cortex (SS-Cx) of the 3xTg-AD mice in the late stage (aged 18 months), which was supported by a failure in autophagy. These results were in contrast to those obtained in the SS-Cx of the CI rats, in which we detected neuronal loss and tauopathy at 1 and 15 days post-ischemia, and this phenomenon was reversed at 30 days. We proposed that this phenomenon was associated with autophagy induction in the late stage, since the data showed a decrease in p-mTOR activity, an association of Beclin-1 and Vps34, a progressive reduction in PHF-1, an increase in LC3B puncta and autophago-lysosomes formation were observed. Furthermore, the survival pathways remained unaffected. Together, our comparative study suggest that autophagy could ameliorates tauopathy in CI but not in AD, suggesting a differential temporal approach to the induction of neuroprotection and the prevention of neurodegeneration.

## Introduction

Cerebral ischemia (CI) is considered the third leading cause of death worldwide (Wen et al., 2004; Zheng et al., 2010), contributing to the development of cognitive decline and dementia, which is induced by a sedentary lifestyle, unhealthy eating habits, diabetes, and other metabolic diseases (Boerma, 2011). It is characterized by an occlusion of the cerebral artery, which results in the deprivation of trophic factors and metabolic substrates, decreased blood flow, and the activation of cell death pathways (Roberts et al., 2013).

Excitotoxicity in CI alters the cellular balance (Arundine and Tymianski, 2004), inducing tau hyperphosphorylation in early- and intermediate-stage post-ischemia (Wen et al., 2004; Cespedes-Rubio et al., 2010). This process is associated with a high incidence of cognitive disorder after stroke (Zheng et al., 2010; Castro-Alvarez et al., 2011). Our previous studies and those of others have demonstrated that a reversal of tauopathy is associated with homeostasis (Kimura et al., 2010; Piedrahita et al., 2010; Castro-Alvarez et al., 2011) as well as with cognitive and motor recovery at 30 and 90 days after an ischemic event (Garrison et al., 2013; Jiang et al., 2013).

Following stroke, three out of four patients develop dementia (Wen et al., 2004), predominantly Alzheimer’s disease (AD; Pluta, 2004). Several studies have demonstrated senile plaques (βA) and amyloid precursor protein (APP) in close proximity to the ischemic focus (Ikeda et al., 2000; Shi et al., 2000), suggesting a degree of convergence in the neuropathogenesis of CI and AD (Jablonski et al., 2011; Song et al., 2013). In stroke pathophysiology, changes in the phosphorylation pattern of the tau protein during and after the ischemic event are observed (Wen et al., 2004). After an infarction, the rapid dephosphorylation of tau occurs, and after blood reperfusion, there is evidence of slow but steady hyperphosphorylation, which causes an accumulation of the tau protein, resulting in long-term brain damage (Wen et al., 2004).

On the other side, patients with AD suffer from a type of dementia belonging to a family of chronic tauopathies, and AD is considered the most common form of dementia worldwide (Schindowski et al., 2006). The intermediate phase of the progression affects the hippocampus and is associated with a loss in short-term memory and behavioral disorders (Walker et al., 2013). The classic hallmarks of AD include amyloid plaques (Aβ) and neurofibrillary tangles (NFTs), which are closely associated with a dramatic neuronal loss (Iqbal and Grundke-Iqbal, 2008) because of the toxic effects generated by cytoplasmic inclusions (Gong et al., 2005; Iqbal and Grundke-Iqbal, 2008; Martin et al., 2011).

Tau is a microtubule-associated protein, which is abundant in axons, promotes polymerization, and modulates the dynamic stabilization of the actin cytoskeleton (Hashiguchi and Hashiguchi, 2013). However, it has been shown that the hyperphosphorilation of tau produces aggregations known as tauopathies (Spillantini and Goedert, 2013; Kimura et al., 2014). A common factor in tauopathies is the aberrant redistribution of tau from the axons to the soma and dendrites and the accumulation of abnormal filaments that are highly phosphorylated and form β-sheets and paired helical filaments (PHFs) prior to the formation of NFTs (Spillantini and Goedert, 2013). This process favors loss of neural circuits that support cognitive dysfunction (Higuchi et al., 2002; Shahani and Brandt, 2002).

Macroautophagy and chaperone-mediated autophagy (CMA) are induced under conditions of cellular stress, including starvation and oxidative stress, or under conditions of altered cellular homeostasis, such as protein aggregation (Singh and Cuervo, 2011). Autophagy plays a crucial role in neurodegeneration (Nixon, 2013). Several studies have shown that autophagy dysfunction contributes to the pathogenesis of several neurological diseases, resulting in an abnormal accumulation of proteins (Murrow and Debnath, 2013), such as in chronic tauopathies (e.g., AD) and acute tauopathies (e.g., CI; Nixon and Yang, 2011; Dohi et al., 2012). However, the mechanisms underlying these pathologies are not clearly understood.

Therefore, in this study, we performed a comparative analysis of two types of tauopathies: (a) chronic injury, such as that found in 3xTg-AD mice model (aged 6, 12, and 18 months) and (b) acute injury such as that found in CI model in rats (1, 15, and 30 days post-ischemia), to identify the role of autophagy on tauopathy induced during a short, intermediate and long-term stages on the progression of both neuropathologies.

## Materials and Methods

### Animal Procedures

The Wistar rats, NoTg mice [3xTg-AD control containing the PS1(M146V) transgene], and 3xTg-AD mice [containing the PS1(M146V), APP(Swe), and tau(P301L) transgenes; Oddo et al., 2003] were bred in-house in a specific pathogen-free (SPF) colony at the vivarium at SIU (Sede de Investigación Universitaria, University of Antioquia, Medellín, Colombia). The animals were maintained on a 12-h: 12-h dark:light cycle and received food and water *ad libitum*. The animals were handled in accordance with the Colombian standards (law 84/1989 and resolution 8430/1993) and NIH guidelines for animal welfare and care (Public Law 99-158, November 20, 1985, “Animals in Research”). Specific care was taken to minimize the animal suffering and to reduce the number of animals used. In total, 60 Wistar rats (aged 3 months, weighing 250–300 g), 30 NoTg mice, and 30 3xTg-AD mice (aged 6, 12, and 18 months, weighing 25–30 g) were used. For the histological and biochemical analyses, five animals per group were used (*n* = 5).

### Global Cerebral Ischemia (2VO)

The animals were anesthetized using ketamine (60 mg/kg) and xylazine (5 mg/kg) and received a 2–4% isoflurane and 96% oxygen mixture via an inhalation anesthesia machine. The body temperature of the animals was monitored using a rectal thermometer throughout the surgery period, and the body temperature was maintained at 33 ± 3°C. A variation of the global cerebral ischemic model was implemented, involving a 2-vessel occlusion (2-VO; Marosi et al., 2006) in which the right common carotid artery (CCA) was permanently occluded and the left CCA was obstructed for 20 min using a vascular clip. After the 20 min, the vascular clip was removed to allow reperfusion. The sham control rats underwent the same procedure without the CCA occlusion. At 24 h following the completion of the surgery, a neurological test was performed to evaluate the sensory and motor abilities of the rats, and a seven-point neurological score was generated (Yrjanheikki et al., 2005). The animals were sacrificed at 1, 15, and 30 days after ischemia for histological and biochemical analyses.

### Histology

The anesthetized animals were perfused with saline buffer and 4% paraformaldehyde (0.1 M phosphate buffer, pH 7.4) using a Varistaltic Pump Plus (Manostat, Barnaut Company) The brains were removed, post-fixed in 4% paraformaldehyde at 4°C for 48 h, and washed with saline buffer. The brains were sectioned into 50-μm slices using a vibrating-blade microtome (Leica VT1000S; Leica Microsystems), cryopreserved using a sucrose gradient (7–30%), and stored at -20°C. The neuronal population and cell morphology were evaluated using Nissl staining with toluidine blue (Sigma) of antero-posterior serial sections for each animal. Briefly, the sections were rinsed in distilled water and immersed in 1% toluidine blue. The sections were dehydrated, immersed in xylene, and mounted using Consultmount®(Thermo). The tissue was visualized using an optical microscope (Nikon, Eclipse E200), and the images were captured using a digital Nikon camera (Sight DS-L1).

### Immunohistochemistry

The sections (sham, *n* = 3; ischemia, *n* = 5) were treated with methanol (50% v/v) and hydrogen peroxide (30% v/v) in 0.1 M phosphate-buffered saline (PBS; pH 7.4) for 20 min to inhibit the endogenous peroxidase and were incubated in 0.1 M phosphate buffer and Triton X-100 (0.5%, v/v) for 30 min. The non-specific antibody binding sites were subsequently blocked using BSA (3%) and Triton X-100 (0.3%, v/v) in 0.1 M PBS for 60 min. The sections were incubated overnight at 4°C in anti-PHF-Tau (AT-8, monoclonal mouse, 1:500; Pierce), which recognizes pSer202/Thr205; PHF-1 monoclonal antibody (a kind gift from P. Davies, Feinstein Institute for Medical Research, Manhasset, NY, USA), which recognizes pSer396/404 in the tau protein; LC3-II polyclonal rabbit antibody (1:250; Cell Signaling Technology); and NeuN monoclonal antibody (1:1,000, Chemicon®). Next, the cells were washed several times in PBS. The sections were incubated with biotinylated mouse and rabbit secondary antibodies (1:250; Pierce) for 90 min at room temperature (RT). The sections were washed four times in PBS, incubated with avidin/biotin peroxidase (1:250; Pierce) for 1 h, and visualized using diaminobenzidine (DAB; Sigma-Aldrich). The sections incubated in the absence of primary antibodies were used as the negative controls. The slides were dehydrated, washed with PBS, and mounted onto coverslips using Consultmount® (Thermo). The tissue sections were visualized using an optical microscope (Nikon, Eclipse E200), and the images were captured using a digital Nikon camera (Sight DS-L1). Intensity of immunoreactivities was determinated by binary system detected by Image J software from NIH.

### Electron Microscopy

Fifty-μm slices from three sham and three ischemic rats perfused with saline buffer and 4% paraformaldehyde (0.1 M phosphate buffer, pH 7.4) were incubated with 0.5% glutaraldehyde in TBS (150 mM NaCl, 30 mM Tris, pH 8.2) for 2 min. Grids were negatively stained for 40 s in uranyl acetate (2%). Samples were examined in a JEM-1010 Transmission Electron Microscope (Jeol, Tokyo, Japan) in the Electron Microscopy Service at the Centro de Biología Molecular Severo Ochoa, Madrid.

### Western Blotting Analyses

The animals were sacrificed, and the cerebral cortices were dissected, immediately frozen in liquid nitrogen, and stored at -80°C prior to use. The samples were lysed in 10 mM Tris (pH 7.4), 100 mM NaCl, 1 mM EDTA, 1 mM EGTA, 10% glycerol, 1% NP40, 1 nM orthovanadate, 5 mM NaF, 1 mM phenylmethylsulfonyl fluoride, and a protease inhibitor cocktail (Sigma-Aldrich; Cardona-Gomez et al., 2004). The lysates (containing ∼30 μg of proteins, quantified using the Bradford method) were loaded onto 8 and 15% electrophoresis gels and transferred onto nitrocellulose membranes (GE Healthcare) at 250 mA for 2 h using an electrophoretic transfer system. The membranes were incubated overnight at 4°C in AT-8 anti-PHF-Tau (rat monoclonal antibody) (1:500; Pierce), rabbit anti-Beclin-1 (1:500; Cell Signaling Technology), rabbit anti-LC3B (1:500; Cell Signaling Technology), rabbit anti-LAMP2-A (1:1,000; Sigma-Aldrich), rabbit anti-mTOR (1:500; Cell Signaling Technology), anti-Vps34 (1:500; Cell Signaling Technology), anti-Hsc70 (1:1,000; Abcam ab137806), anti-HSP90 (1:1000; Santa Cruz Biotechnology Inc.), anti-CHIP (1:1,000; Sigma-Aldrich), PHF-1 monoclonal antibody (Donated by P. Davies, Feinstein Institute for Medical Research, Manhasset, NY, USA), and mouse anti-β actin and anti-tubulin (1:4,000; Promega). IRDye 800CW goat anti-mouse or anti-rabbit (LI-COR; 1:5,000) and anti-mouse IgG or anti-rabbit IgG peroxidase-conjugated antibodies (Pierce Biotechnology; diluted 1:10,000) were used as the secondary probes. The blots were developed using the Odyssey Infrared Imaging System. To minimize interassay variation, samples from all of the experimental groups were processed in parallel.

### Immunoprecipitation

The animals (*n* = 5 for each experimental group) were sacrificed by decapitation, and the brains were quickly removed. The cerebral cortices obtained from the 3xTg-AD mice and sham- and post-ischemic rats were dissected and frozen at -80°C until further analysis. The samples were homogenized in lysis buffer containing 150 mM NaCl, 20 mM Tris (pH 7.4), 10% glycerol, 1 mM EDTA, 1% NP40, 100 μM phenylmethylsulfonyl fluoride, 1 μg/ml aprotinin and leupeptin (Sigma), and 100 μM orthovanadate. The lysates were clarified by centrifugation at 14,000 rpm for 5 min. A protein assay was performed, and 250 μg of protein was incubated overnight at 4°C in the presence of Beclin-1 or Vps34 antibodies (1:250, Cell Signaling Technology). Protein G-sepharose beads were added, and the samples were incubated for an additional 2 h at RT. The immune complexes were washed three times using an immunoprecipitation lysis buffer prior to the SDS-PAGE and immunoblotting. The proteins were separated using 10% SDS-PAGE, transferred onto nitrocellulose membranes (Amersham), and probed with Beclin-1 (1:1,000; Cell Signaling) or Vps34 antibodies (1:500; Calbiochem). The lysates were used as the positive controls, and incubation with IgG peptide was used as the negative immunoprecipitation control.

### mTOR Kinase Activity

The K-LISA mTOR Activity Kit (Merck/Millipore) was used to measure the mTOR kinase activity. The cerebral cortical lysates obtained from the 3xTg-AD mice and ischemic rats and their respective controls (NoTg mice and sham rats, respectively) were homogenized in 50 mM Tris-HCl (pH 7.4), 100 mM NaCl, 50 mM β-glycerophosphate, 10% glycerol, Tween 20, 1 mM EDTA, microcistin LR, 20 mM NaF, and 25 mM protease inhibitor cocktail. The samples were clarified of insoluble material by centrifugation at 20,000 rpm for 10 min at 4°C. The mTOR primary antibody (6 μg) was added to the supernatants and incubated for 1 h at 4°C. Next, G Sepharose beads were added, and the samples were incubated with stirring for 60 min at 4°C and centrifuged at 4,000 rpm for 5 min at 4°C. The pellet was washed using 1X kinase assay buffer and incubated in 2X assay buffer and the mTOR kinase substrate WS (working solution) for 30 min at 30°C. The enzymatic reaction was terminated using the kinase stop solution, and the supernatant was added to the glutathione-coated wells and incubated for 60 min at 30°C, followed by two washes and incubation for 1 h at RT with 100 μl anti-WS p70S6K-T389. Next, the samples were washed three times using 100-μl HRP secondary antibody for 1 h. Finally, the colorimetric reaction was stopped using ELISA stop solution, and the absorbance was read at 450 nm. The colorimetric values reflected the amount of mTOR kinase activity.

### Immunofluorescence

The mouse and rat brains were fixed in 4% paraformaldehyde in PBS, cryopreserved with 30% sucrose, and stored at 20°C. The brains were cut into 50-μm coronal sections using a vibratome (Leica 1000) and treated with 50 mM NH4Cl for 10 min at RT. The tissue sections were preincubated for 1 h in 1% BSA containing 0.3% Triton X-100 in 0.1 M P and incubated with primary antibodies, polyclonal anti-LC3B (1:200, Cell Signaling Technology) and monoclonal PHF-1 overnight at 4°C. The secondary antibodies were conjugated to Alexa 594 and 488 fluorophores (Molecular Probes). The sections were visualized using a Confocal-DSU Olympus IX-81 microscope and analyzed as individual images for LC3, Hoechst, and PHF-1 immunofluorescence. The deconvolution, maximal projection, and fluorescence intensity were determined using the Image Scope-Pro software (Media Cybernetics) and Cell software (Olympus).

### Statistics Analysis

In each experimental group, five animals were histologically and biochemically analyzed (*n* = 5, represents the number of animals used for the statistical analyses). The homogeneity of the variance test was applied prior to the statistical analysis. Parametric data were compared using one-way analysis of variance (ANOVA) with Tukey’s *post hoc* test to compare several independent groups. The Fisher *Z*-test was used to normalize the values, and the data were analyzed using ANOVA with Tukey’s *post hoc* tests. The analyses were performed using SPSS 18.0 and GraphPad Prism version 5.00 software (University of Antioquia license). The data were expressed as the mean ± the SEM with *p* < 0.05^∗^ and *p* < 0.01^∗∗^. To diminish interassay variation, all of the sample groups were processed in parallel. In addition, the data obtained for the 3xTG-AD mice were analyzed and compared with the average value of the internal control for the NoTg mice for each age group (6, 12, and 18 months). The ischemic rats were compared to the sham rats for each assay and at each post-ischemia time point (1, 15, and 30 days).

## Results

### Comparative Analysis of Neuronal Loss in the Primary Somatosensory Cortex of 3xTg-AD Mice and Global Cerebral Ischemic Rats

**FIGURE 1:**
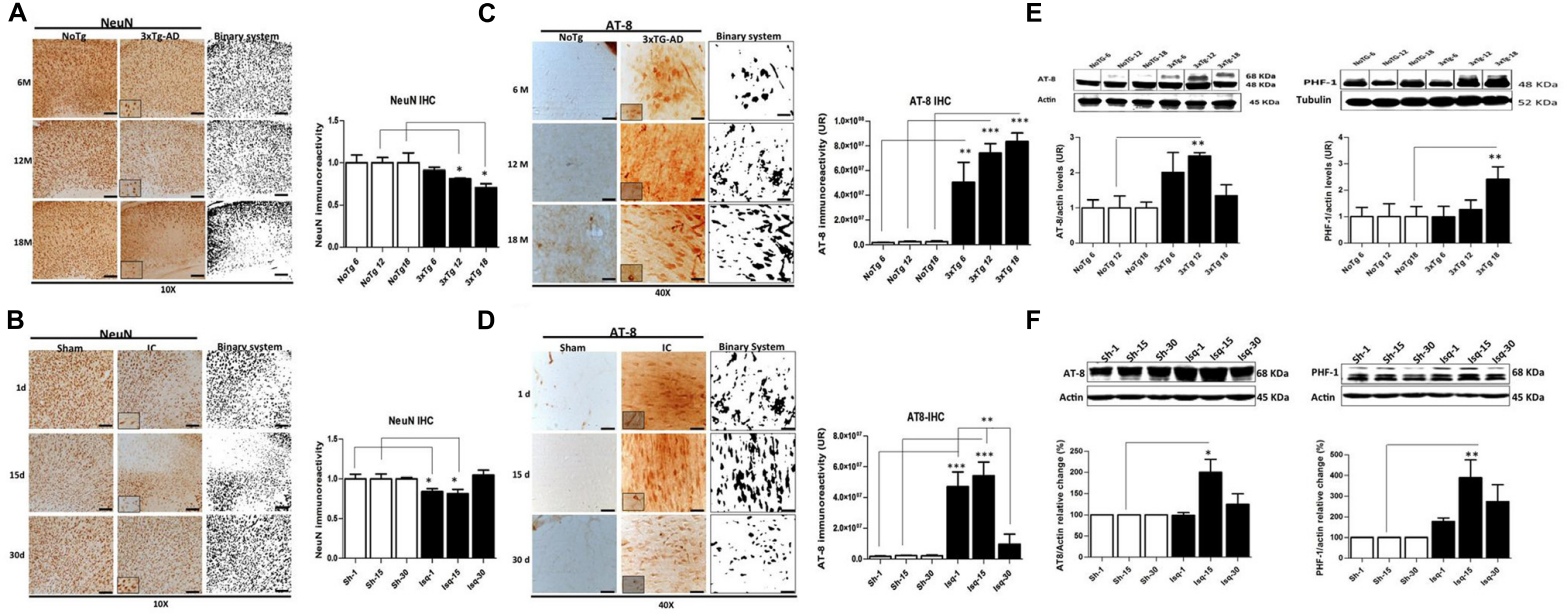
**Temporal course of the neuronal population and hyperphosphorylated tau in 3xTg-AD mice and 2VO cerebral ischemia in rats. (A)** NeuN immunoreactivity (IR) decreased at 6 (*p* ≤ 0.0212), 12 (*p* ≤ 0.0213), and 18 months (*p* ≤ 0.0422) in the SS-Cx of the 3xTgAD mouse model. **(B)** NeuN IR decreased at 1 (*p* ≤ 0.0403) and 15 days (*p* ≤ 0.0417) post-ischemia, while increased at 30 days post-ischemia (*p* ≤ 0.2616). Both models were compared with the internal control groups, NoTg and sham animals respectively *n* = 3 animals/group. Scale bar, 150 μm; magnification 10X (insert 100X). **(C)** AT-8 IR increased at 12 months (*p* ≤ 0.0001) and 18 months (*p* ≤ 0.0001) in the SS-Cx of the 3xTgAD mice compared with the 3xTg-AD mice at 6 months (*p* ≤ 0.001) and NoTg mice. **(D)** AT-8 IR increased at 1 (*p* ≤ 0.0001) and 15 days (*p* ≤ 0.0001) post-ischemia in rats, while it was decreased at 30 days post-ischemia (*p* ≤ 0.1388), compared with the sham rats. Magnification, 40X (insert 100X); *n* = 4–5. **(E)** Western blots showing representative bands of AT-8 and PHF-1 increased at 12 (*p* ≤ 0.001) and 18 (*p* ≤ 0.02) months in SS-Cx total lysates from 3xTg AD. **(F)** AT-8- (*p* ≤ 0.011) and PHF-1 (*p* ≤ 0.020) protein levels increased at 15 days post-ischemia compared with the internal controls. β-actin was used as the loading control. The data are expressed as the mean ± SEM; *n* = 4–5; ^∗^*p* < 0.05; ^∗∗^*p* < 0.01; ^∗∗∗^*p* < 0.0001.

Neuronal loss is a major neuropathological indicator used to measure the degree of alteration in nervous tissue (Buchman et al., 2013) and has been previously demonstrated in both AD and CI (Cespedes-Rubio et al., 2010; Abuhassan et al., 2011). We analyzed the temporal course of neuronal nuclei immunoreactivity (NeuN; Mullen et al., 1992; Wolf et al., 1996) in the somatosensory cortex, as common affected area during acute and chronic injury, during the short-term, intermediate, and long-term stages of disease in the 3xTg-AD and global CI (2-VO) models. We observed changes in NeuN immunoreactivity levels in the 3xTG-AD mice at 6 months of age (4,488 ± 177 nuclei) compared with the NoTg mice (4,886 ± 460 nuclei). However, a significant decrease in the NeuN immunoreactivity levels was observed at 12 months (3,787 ± 43 nuclei) and 18 months (3,319 ± 222) compared with the control group at these ages (4,662 ± 294 and 4,701 ± 562 cores, respectively; **Figure 1A**). These data confirmed a progressive neuronal loss in AD, mainly during the late stages of the disease (Hubbard and Anderson, 1985; Scheff and Price, 2006; Padurariu et al., 2012; Reijmer et al., 2013).

In the global CI rat model, the penumbral area in the somatosensory cortex, which is closest to the focus in the region of the middle cerebral artery, demonstrated a significant reduction in the NeuN immunoreactivity (**Figure 1B**) at day 1 (2,322 ± 109 nuclei) and day 15 (2,531 ± 185 nuclei) post-ischemia compared with the control rats (2,780 ± 158 and 3,395 ± 206, respectively). Interestingly, the NeuN immunoreactivity recovered at 30 days post-ischemia (2,990 ± 179 nuclei) compared to the internal control group (2,858 ± 61 nuclei; **Figure 1B**). These results confirmed previous findings demonstrating a neuronal loss in the somatosensory cortex during the acute phase post-ischemia (Guadagno et al., 2008; Kiran, 2012; Carrera et al., 2013).

### Hyperphosphorylated tau Progressively Increases in the Somatosensory Cortex of 3xTg-AD Mice but is Reversed in the Late Stage in Ischemic Rats

The aggregation of hyperphosphorylated tau protein has been described as one of the main histopathological hallmarks that lead to neuronal cell death and promote neurodegenerative diseases and dementia (Kosik and Shimura, 2005). We evaluated the temporal course of phosphorylated tau using AT-8 and PHF-1 antibodies in short-term, intermediate, and long-term disease progression in the somatosensory cortex of chronic injuries, such as in AD (at 6, 12, and 18 months) and in acute injury, such as CI (2VO at 1, 15, and 30 days post-ischemia). Our data show increased AT-8 immunoreactivity in the 3xTg-AD mice at 12 and 18 months compared with the 3xTg-AD mice at 6 months and the respective controls (NoTg; **Figure 1C**). These observations were supported by the increased AT-8 protein levels at 12 months and PHF-1- protein levels at 18 months in the 3xTgAD mice compared with the internal controls for each age group (**Figure 1D**), confirming the direct relationship between tauopathy and disease progress, as previously described (Oddo et al., 2003). In addition, these findings confirmed the inverse relationship of tauopathy with neuronal loss in an advanced stage of the neuropathology (aged 12 and 18 months).

Compared with the ischemic rats, the levels of AT-8 immunoreactivity were increased at 1 and 15 days post-ischemia. However, these levels were significantly decreased at 30 days after stroke compared with the internal control (**Figure 1E**). These observations were supported by the statistically significant increase in the AT-8- and PHF-1 protein levels at 15 days post-ischemia compared with the internal control (**Figure 1F**). These findings demonstrated early tauopathy induced by acute injury (1 and 15 days) via global CI (2VO), which was reversed at 30 days.

### mTOR Kinase Activity is Upregulated in the Late Stage Progression in 3xTgAD Mice and Downregulated in the Late Stage Progression in Cerebral Ischemic Rats

**FIGURE 2:**
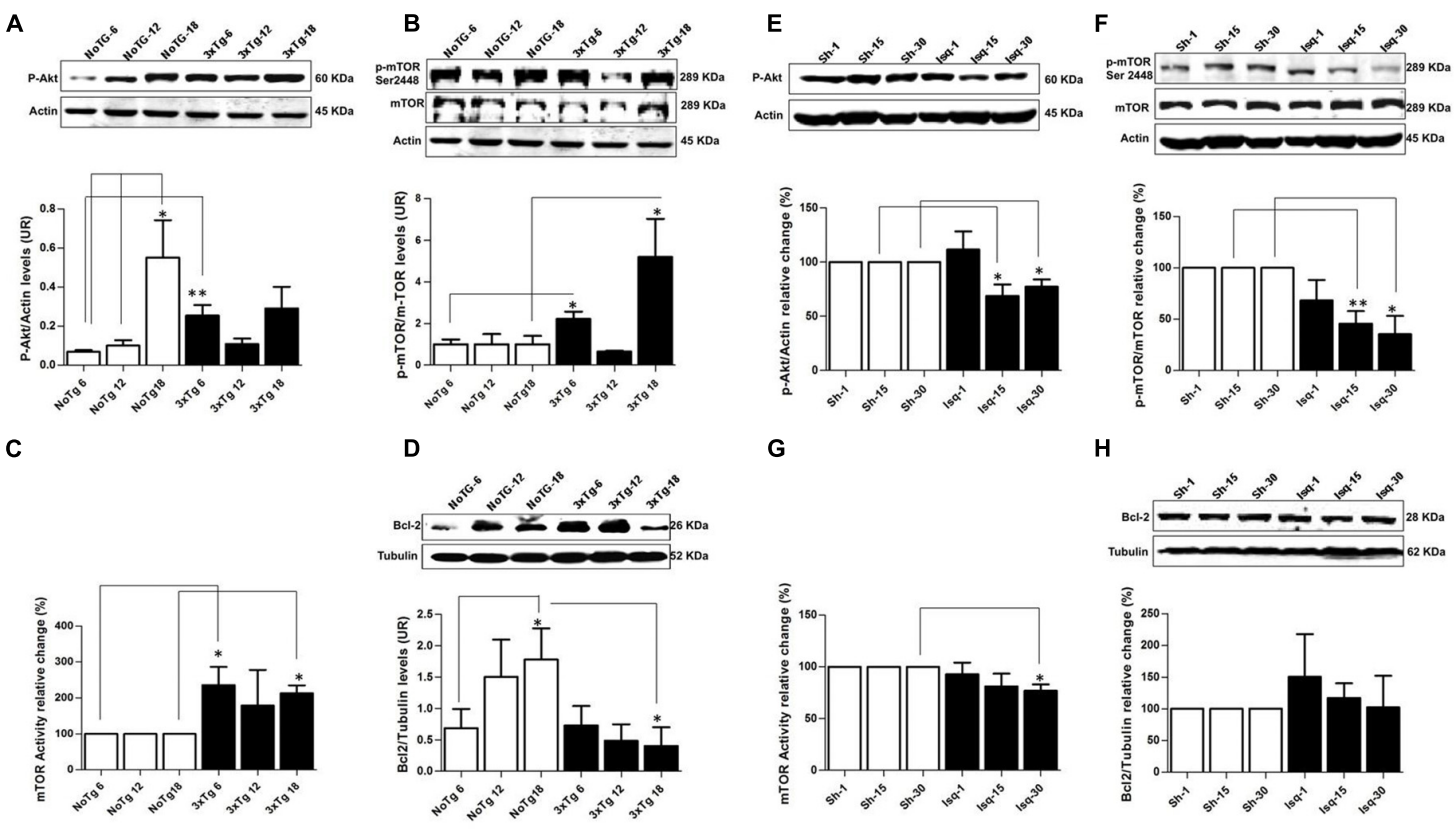
**Altered p-AKT/p-mTOR activation in the cerebral cortex of 3xTgAD mice compared with cerebral ischemia in rats.** Representative bands from SS-Cx lysates of 3xTgAD and ischemic rats were evaluated by Western blotting. **(A)** p-Ser473 Akt protein levels increased in NoTg mice at 18 months (*p* ≤ 0.005) compared with t NoTg mice at 6 and 12 months. Also, p-Ser473 Akt protein levels increased at 6 months in 3xTg-AD (*p* ≤ 0.0045). **(B)** p-Ser2448 mTOR protein levels increased at 6 (*p* ≤ 0.0150) and 18 (*p* ≤ 0.0339) months in 3xTgAD mice, **(C)** mTOR activity increased at 18 months (*p* ≤ 0.017), **(D)** Bcl-2 protein levels Increased in NoTg mice at 18 months (*p* ≤ 0.045), and there was a reduction in the levels of Bcl-2 in the 3xTg-AD mice at 18 months (*p* ≤ 0.038). **(E)** p-Ser473 Akt decreased at 15 days (*p* ≤ 0.0258) and 30 days (*p* ≤ 0.0147) post-ischemia, **(F)** similarly p-Ser2448 mTOR decreased at 15 days (*p* ≤ 0.0056) and 30 days (*p* ≤ 0.0183) after cerebral ischemia. **(G)** mTOR kinase activity decreased at day 30 post-ischemia (*p* ≤ 0.0330). **(H)** No significant changes were detected in the Bcl-2 protein levels in any post-ischemia time point. The data were normalized against the internal control for each time point for the NoTg mice **(A–D)** and sham rats **(E–H)**. β-actin and β-III tubulin were used as the loading controls. The data are shown in the bar graph as arbitrary units (AUs). The data are presented as the mean ± SEM; *n* = 3–5; ^∗^*p* < 0.05; ^∗∗^*p* < 0.001.

The pAkt/mTOR pathway is involved in the control of autophagy (Spilman et al., 2010; Chong et al., 2012). In this study, we evaluated the relationship between the activation of the mTOR pathway and tau hyperphosphorylation. We determined that the p-Ser473 Akt protein levels but not the p-Ser2448 mTOR protein levels were significantly increased in the NoTg animals at 18 months compared with the NoTg mice at 6 and 12 months. However, there was a significant increase in p-Ser473 Akt (**Figure 2A**) and p-Ser2448 mTOR at 6 months (**Figure 2B**) as well as an increase at 18 months in the 3xTg-AD mice (**Figure 2B**). These findings were clearly supported by the increase in mTOR activity at 6 and 18 months without any significant changes at 12 months in the 3xTg-AD mice compared with the age-matched NoTg control mice (**Figure 2C**). Increased levels of Bcl-2, which acts downstream of the Akt/mTOR pathway, were observed in the NoTg mice at 18 months, even in the absence of changes in the level and activity of p-Ser2448 mTOR (**Figure 2D**). Nevertheless, there was a significant reduction in the levels of Bcl-2 in the 3xTg-AD mice at 18 months (**Figure 2D**), and no changes were observed at the other time points compared with the control groups. These findings indicated that the NoTg mice demonstrated an increase in the level of Akt/Bcl-2 proteins associated with age. However, the 3xTgAD mice demonstrated a defective pathway, as indicated by the increase in mTOR kinase activity and the significant reduction in Bcl-2 levels at the late stage of the disease.

In contrast, the ischemic rats demonstrated a significant decrease in p-Ser473 Akt and p-Ser2448 mTOR protein levels at 15 and 30 days after CI, respectively (**Figures 2E,F**), which was supported by the significant downregulation in mTOR kinase activity at day 30 in the ischemic group compared with the sham rats (**Figure 2G**). Finally, no significant changes were detected in the Bcl-2 protein levels in any of the post-cerebral ischemic time points compared with the controls (**Figure 2H**). These findings suggested that the low levels of p-Akt and mTOR kinase activity and the maintenance of basal levels of Bcl-2 promoted cellular homeostasis at 30 days post-ischemia, which was consistent with the neuronal population recovery and decrease in hyperphosphorylated tau.

### Macroautophagy-Associated Proteins Decreased in the Late Stage of 3xTg-AD, but were Induced in Late Stage of Cerebral Ischemia

**FIGURE 3:**
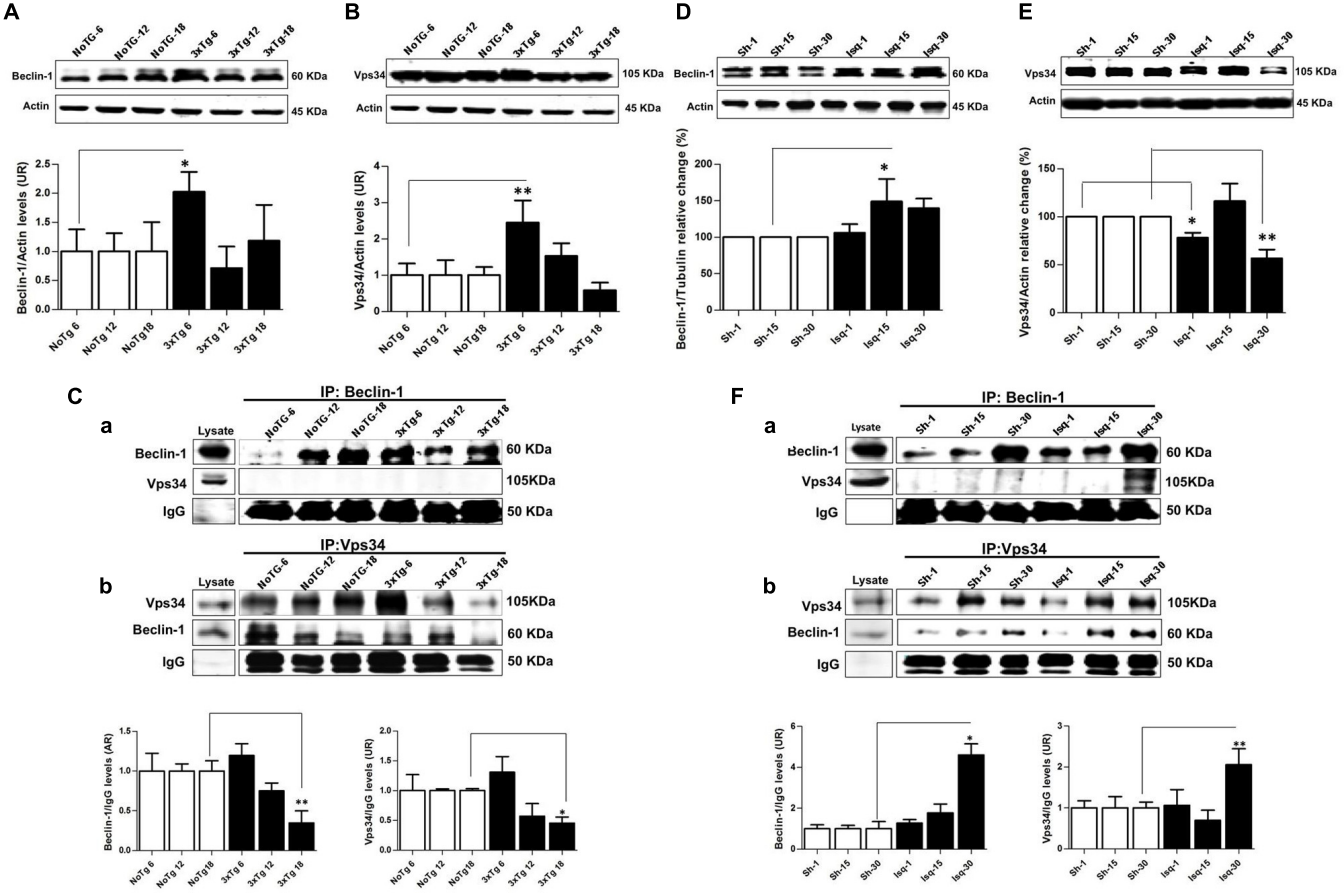
**Macroautophagy effects on the cerebral cortex of 3xTgAD mice compared with cerebral ischemia in rats.** Representative bands from SS-Cx lysates of 3xTgAD and ischemic rats were evaluated by Western blotting and immunoprecipitation. **(A)** Beclin-1 (*p* ≤ 0.043) and **(B)** Vps34 (*p* ≤ 0.029) increased in 3xTg-AD at 6 months. **(C)** The Vps34/Beclin-1 association was lost at 12 and 18 months. **(D,E)** Beclin-1 increased at 15 days in Ischemic rats (*p* ≤ 0.029) and Vps34 was reduced at 1 day (*p* ≤ 0.021) and 30 days (*p* ≤ 0.007). **(F)** Association between Beclin-1 and Vps34 was detected at 30 days and Vps34/Beclin-1 complex formation at 15 and 30 days post-ischemia. The data from the 3xTgAD mice and ischemic rats were normalized against the internal control for each time point in the NoTg mice and sham rats, respectively. β-actin was used as the loading control. The data are shown in the bar graph as AUs. IgG was used as the loading control in the immunoprecipitation assay. The data are presented as the mean ± SEM; *n* = 3–5; ^∗^*p* < 0.05; ^∗∗^*p* < 0.001.

**FIGURE 4:**
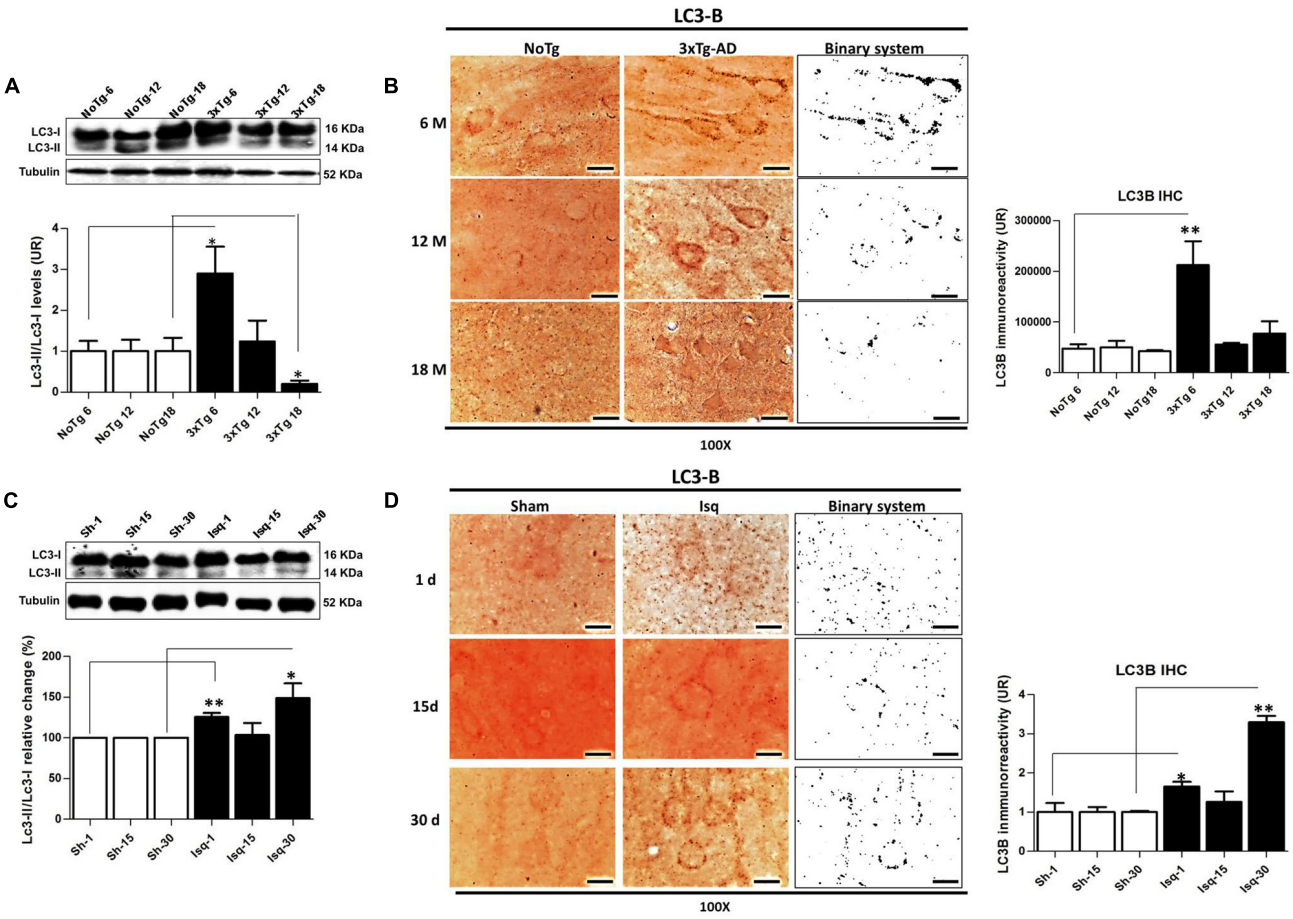
**LC3B immunoreactivity in the cerebral cortex of 3xTgAD mice and in cerebral ischemic rats. (A)** Increase in the LC3B-II/LC3B-I ratio (*p* ≤ 0.010) and **(B)** LC3B puncta formation at 6 months (*p* ≤ 0.0013) in 3xTg-AD. **(C)** The LC3B-II/LC3B-I ratio was increased on day 1 (*p* ≤ 0.0239) and at 30 days post-ischemia (*p* ≤ 0.0032). **(D)** LC3B immunoreactivity puncta formation was increased at 1 (*p* ≤ 0.0125) and 30 days (*p* ≤ 0.0058) post-ischemia. The bands are representative of LC3B-I and LC3B-II in AD and CI, respectively. LC3B-I and LC3B-II protein levels and ratios were evaluated using Western blots. β-actin was used as the loading control *n* = 5–6. The data are shown as AUs. LC3B immunoreactivity was converted into a binary system for densitometric analyses. An LC3B puncta pattern was observed. Magnification, 100X; scale bars, 5 μm; *n* = 3. The data were compared to the internal control values for each time point in the disease progression. The data are expressed as the mean ± SEM; ^∗^*p* < 0.05; ^∗∗^*p* < 0.001.

The Beclin-1 and Vps34 proteins are crucial for the assembly of the phagophore assembly site (PAS; Funderburk et al., 2010), and LC3B is an autophagosomal marker (Kang et al., 2011) that is associated with autophagic flux (Rubinsztein, 2012). To determine the role of macroautophagy in the tauopathy generated after acute (CI) and chronic (3xTg-AD) injury, we performed a comparative analysis of these proteins in both models. We found that Beclin-1 and Vps34 increased at the early stage of progression (6 months) in the 3xTg-AD mice (**Figures 3A,B**) compared with the controls. Although a Vps34/Beclin-1 association was observed at 6 months, this association was not reciprocally confirmed in the detection of the inverse Beclin-1/Vps34 complex (**Figure 3C**). The Vps34/Beclin-1 association was progressively lost at 12 and 18 months and was significantly reduced in the late stage progression (18 months) in the 3xTg AD mice (**Figure 3C**). In addition, these data were supported by the increase in the LC3B-II/LC3B-I ratio (**Figure 4A**) and LC3B puncta formation at 6 months (**Figure 4B**). However, a progressive decrease in the LC3B-II/LC3B-I ratio was observed at 18 months without any changes in the LC3B puncta compared with the controls (**Figure 4B**) in the somatosensory cortex of the 3xTg-AD mice.

**FIGURE 5:**
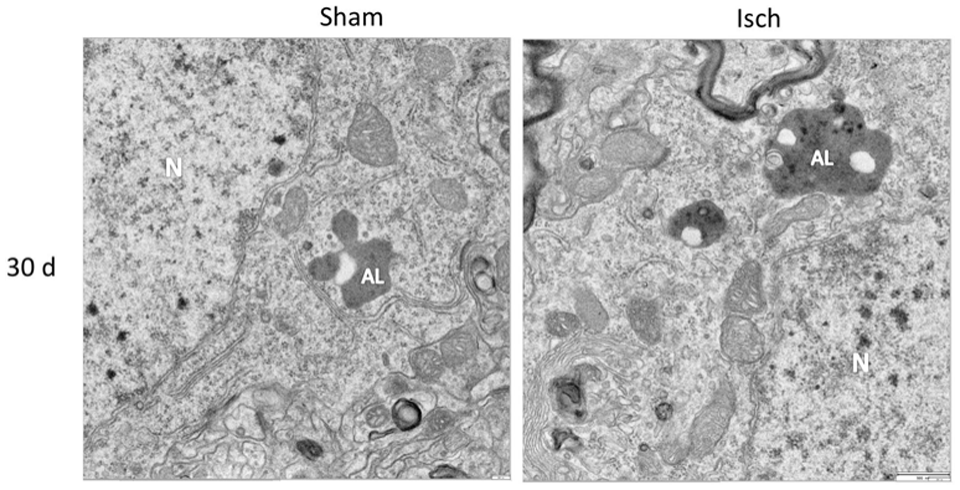
**Autophago-lysosomes at 30 days post-ischemia.** Detection of Autophago-lysosomes (ALs) and bubble-like (BL). Representative images obtained from SS-Cx cortex of sham and ischemic rats at 30 days post-stroke by TEM.

Nevertheless, there was a significant increase in Beclin-1 at 15 days in the somatosensory cortex of the ischemic rats (**Figure 3D**), although Vps34 was significantly reduced at 1 and 30 days and did not change at 15 days after ischemia (**Figure 3E**). However, a significant association between Beclin-1 and Vps34 was detected at 30 days, which was supported by the Vps34/Beclin-1 complex formation at 15 and 30 days post-ischemia (**Figure 3F**). The LC3B-II/LC3B-I ratio was increased on day 1 and at 30 days post-ischemia (**Figure 4C**), which support the LC3B immunoreactivity puncta distribution was significantly increased at 1 and 30 days post-ischemia (**Figure 4D**) compared with the internal controls. These data demonstrated a major variation at 1 day post-ischemia with an apparent lack of PAS formation (Beclin-1/Vps34), but an increase in LC3B-positive autophagosomes. Although PAS and LC3B puncta were detected at 30 days after stroke, there was a concomitant reduction in hyperphosphorylated tau at this late stage post-ischemia (**Figure 1E**). Which could be supported by detection of bigger autophago-lysosome (ALs; 36/106 total ALs, 35%) and bubble-like (BL; 64/129 total ALs, 49%) on cerebral cortex of ischemic rats at 30 days, compared respect to the detection of AL (20/115 total ALs, 17%) and BL (21/101 total Als, 20 %) on cerebral cortex from sham rats analyzed by transmission electron microscopy (**Figure 5**).

### LC3B Immunofluorescence is Increased When Hyperphosphorylated tau is Reduced in Late-Stage Cerebral Ischemia in Rats but is Decreased in the Tauopathy in 3xTg-AD Mice

**FIGURE 6:**
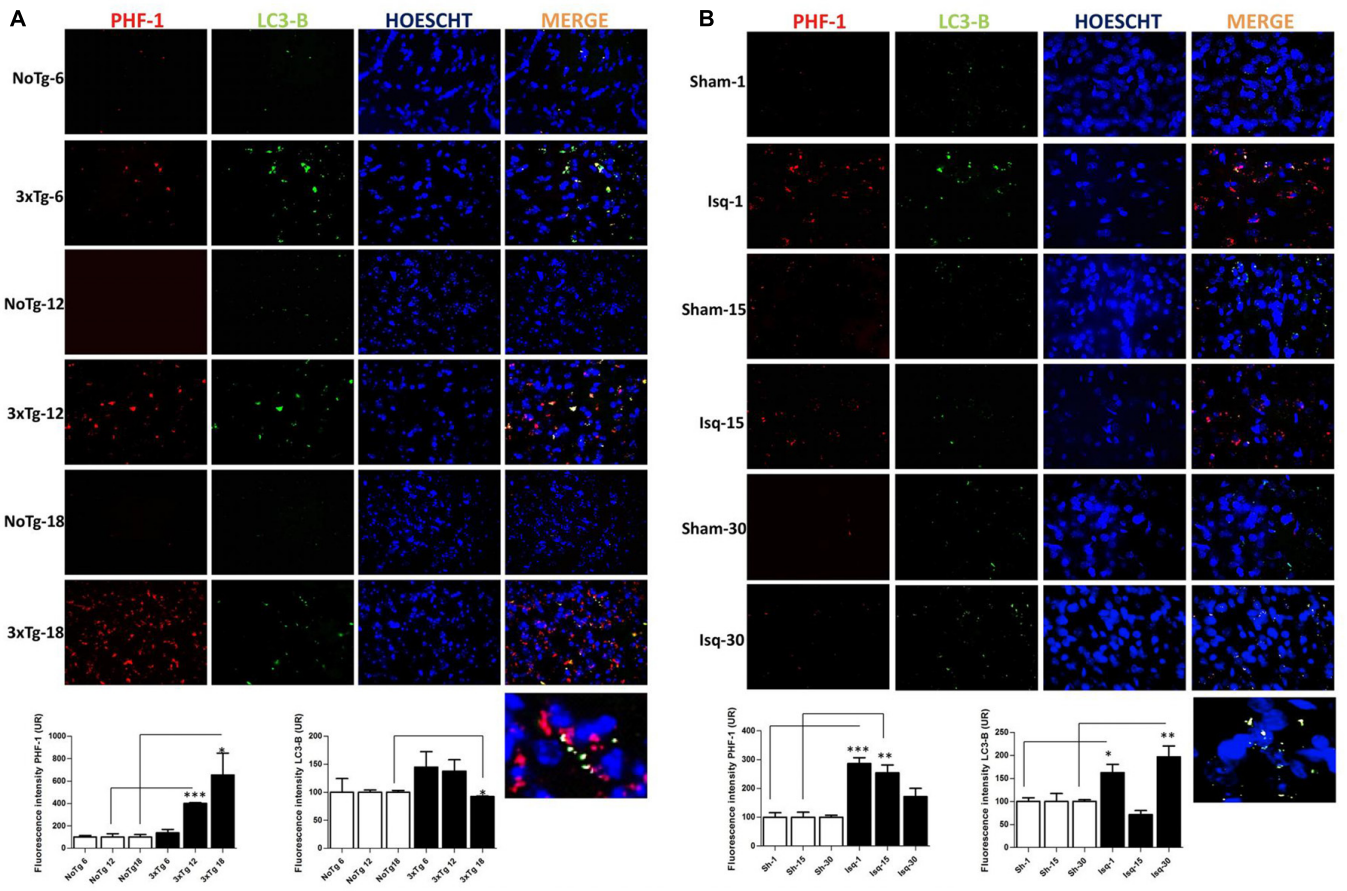
**LC3B and PHF-1 immunofluorescence in the cerebral cortex of 3xTgAD mice and in cerebral ischemic rats.** LC3B and PHF-1 immunofluorescence was detected in the SS-Cx of 3xTgAD mice and cerebral ischemic rats. **(A)** PHF-1 fluorescence intensity increased in 3xTg-AD mice at 12 (*p* ≤ 0.0003) and 18 months (*p* ≤ 0.0233), and LC3B fluorescence intensity decreased in 3xTg-AD mice at 18 months (*p* ≤ 0.0499). **(B)** PHF-1 fluorescence intensity increased at 1 (*p* ≤ 0.0001) and 15 days post-ischemia (*p* ≤ 0.0079), and a reduction at 30 days (*p* ≤ 0.1191) post-ischemia, and LC3B fluorescence intensity increased at 1 day (*p* ≤ 0.0156) and 30 days post-ischemia (*p* ≤ 0.0077). The data were compared with the internal control values for each time point in the disease progression. Magnification, 60; scale bar, 20 μm; *n* = 3. The data were expressed as the mean ± SEM; ^∗^*p* < 0.05; ^∗∗^*p* < 0.004; ^∗∗∗^*p* < 0.001.

To confirm the interdependency between the levels of hyperphosphorylated tau and macroautophagy, we evaluated the immunofluorescence intensity of PHF-1 and LC3B in the primary somatosensory cortex in the short, intermediate, and long-term progression of chronic injury (3xTg-AD at 6, 12, and 18 months, respectively) and acute injury (CI – 2VO at 1, 15, and 30 days post-ischemia). We observed a significant increase in the fluorescence intensity of PHF-1 immunoreactivity in the 3xTg-AD mice at 12 and 18 months compared with the internal controls at each time point. Interestingly, we observed a significant reduction in the fluorescence intensity of LC3B in the 3xTg-AD mice at 18 months (**Figure 6A**). Taken together, these findings suggested that formation of autophagosomes is altered, since LC3B is decreased, which could favor the Tau aggregation in advanced stages of chronic injury, such as in the 3xTg-AD model mice.

However, when we assessed the somatosensory cortex of the ischemic rats, we found a significant increase in the PHF-1 fluorescence intensity at 1 and 15 days post-ischemia, which was reversed on day 30 post-ischemia, compared with the controls for each time point. Interestingly, we observed an up-regulation in the fluorescence intensity of LC3B at 1 and 30 days post-ischemia (**Figure 6B**) compared with the temporal course of the sham animals. These results were consistent with our previous data, suggesting that autophagosomes formation were increased at late stage post-ischemia, which could be associated with decline of the hyperphosphorylated tau aggregates in the same stage.

### Hsc70 and LAMP2 are Differentially Affected in Chronic and Acute Tauopathies

**FIGURE 7:**
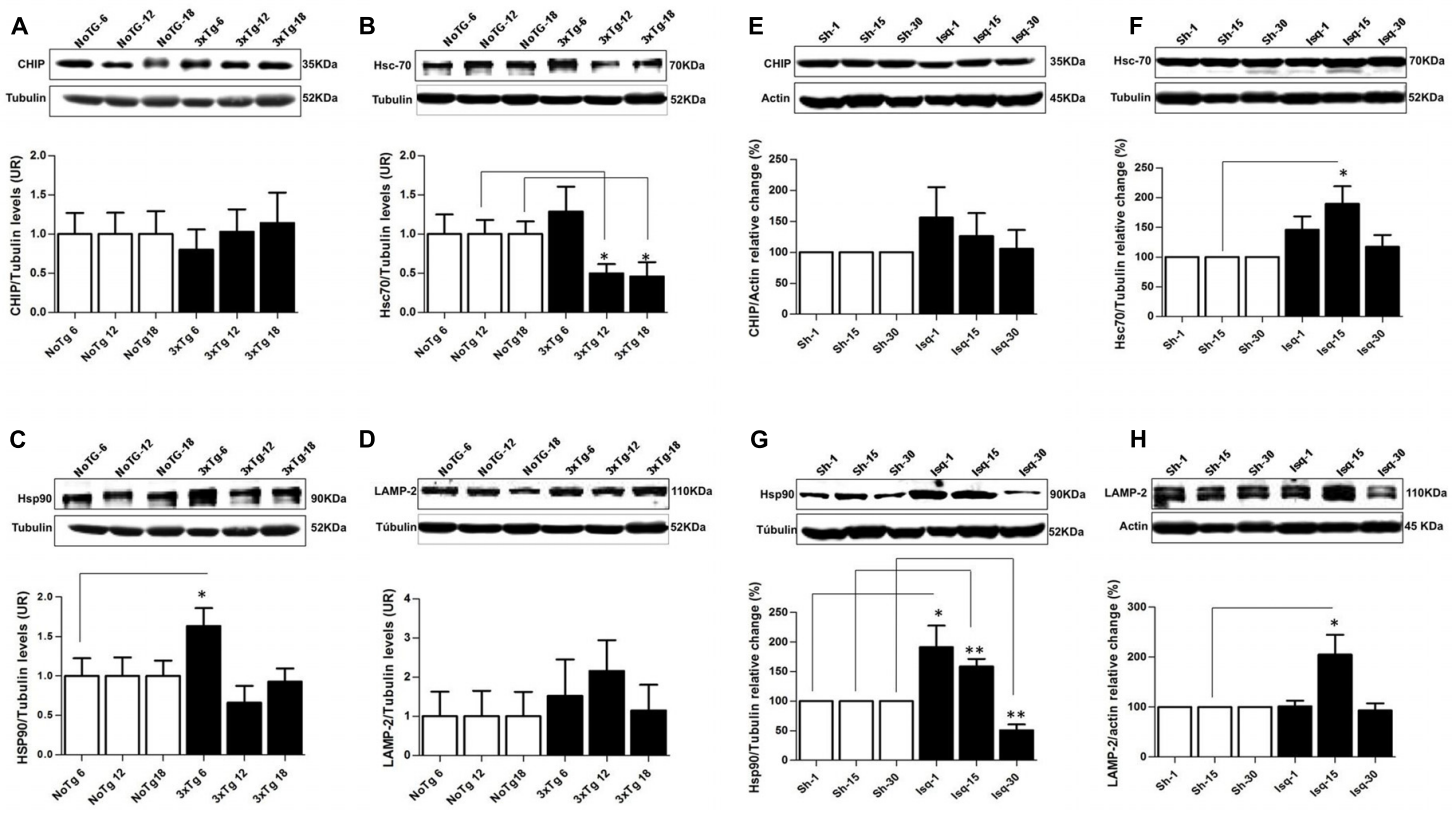
**Proteins related to CMA and homeostasis are differentially affected in AD and CI.** Representative bands from SS-Cx lysates of 3xTgAD and ischemic rats were evaluated by Western blotting **(A)** CHIP was unmodified, **(B)** Hsc70 decreased at 12 months (*p* ≤ 0.302) and at 18 months (*p* ≤ 0.034), **(C)** Hsp90 increased at 6 months (*p* ≤ 0.0409), **(D)** LAMP-2 levels were unaltered in the 3xTg-AD mice. **(E)** CHIP was unmodified, **(F)** Hsc70 and **(H)** LAMP2 increased at the intermediate stage (15 days) post-stroke (*p* ≤ 0.05). **(G)** Hsp90 levels increased at 1 (*p* ≤ 0.0432) and 15 days (*p* ≤ 0.0093) and decreased at 30 days (*p* ≤ 0.0076) after ischemia. The data were normalized against the internal control for each time point in the NoTg mice and sham rats, respectively. β-III tubulin and actin were used as the loading controls. The data are shown in the bar graph as AUs. The data were compared to the internal control values for each time point in the disease progression. The data are presented as the mean ± SEM; *n* = 3–5; ^∗^*p* < 0.05; ^∗∗^*p* < 0.001.

Chaperone-mediated autophagy demonstrates an interdependency with macroautophagy (Kaushik et al., 2008) in the promotion of homeostasis (Kon and Cuervo, 2010). Therefore, we analyzed several CMA-related proteins in the context of tauopathy progression. Our data showed that the chaperone-assisted ubiquitin ligase (CHIP) was not modified (**Figure 7A**), Hsc70 was down-regulated at 12 and 18 months in the 3xTg-AD mice (**Figure 7B**) and that Hsp90 was increased at 6 months, but there were not changes in these protein levels at 12 and 18 months (**Figure 7C**). LAMP2 (lysosome membrane marker) protein levels were unaltered compared to the controls (**Figure 7D**). These results suggested a negative effect on the CMA pathway, where low Hsc70 levels were used as an indicator of an alteration at late-stage AD in the 3xTg-AD mice.

In the somatosensory cortex of the ischemic rats, we demonstrated that CHIP was unaltered at the different stages of the disease (**Figure 7E**). Moreover, the Hsc70 and LAMP2 protein levels demonstrated an up-regulation at the intermediate stage (15 days) post-stroke, although these levels remained unchanged at 30 days (**Figures 7F,H**). And the Hsp90 levels were increased at 1 and 15 days and significantly reduced at 30 days after ischemia (**Figure 7G**). These findings suggested that in an intermediate time point post-ischemia the CMA pathway could be induced, favoring the degradation of tau hyperphosphorylated, which was consistent with higher levels of Hsp90, Hsc70, and LAMP2. Moreover, hyperphosphorylated tau was reversed at 30 days after stroke when the macroautophagy markers were increased.

## Discussion

Our study present by first time a comparative analysis of chronic and acute tauopathies in animal models. Our results suggested that there was an increase of autophagy-related markers, coincident with the inhibition of the Akt/mTOR pathway at late-stage (30 days) post-ischemia, when reduced the tauopathy and decreased neuronal loss in the CI model. However, these changes were not observed at the late-stage (18 months) of 3xTg-AD progression, favoring the formation of NFTs and cerebral impairment. These findings suggest a time window for neuroprotective therapy for the induction of autophagy.

AD and CI are neurological disorders characterized by protein aggregation and neuronal death, which results in cognitive and behavioral disorders (Wen et al., 2004; Golde and Miller, 2009; Shelkovnikova et al., 2012). Tauopathy increases progressively with age in AD animal models (Oddo et al., 2003) and in the brains of AD patients (Diniz et al., 2008). The sustained upregulation of various kinases, such as CDK5 kinase, JNK, MAPK, and GSK3 (Planel et al., 2004), results in the formation of PHFs and NFTs (Alonso et al., 1996) and leads to a disruption in axonal transport (Grundke-Iqbal et al., 1986; Schindowski et al., 2006; Iqbal and Grundke-Iqbal, 2008).

Akt and mTOR are essential homeostatic components that are altered during chronic (AD) or acute (CI) neurological disease (Meske et al., 2008; Chong et al., 2012). Akt is a pro-survival protein that is triggered by cell stress during aging (Chong et al., 2005) and by downstream effectors that upregulate Bcl-2 (Levine et al., 2008), which was confirmed in our study in the oldest NoTg mice. In addition, activated Akt was detected at the earliest stage (6 months) of the chronic lesion in the 3xTg-AD model, which may be because of compensation (Datta et al., 1999). However, recent evidence has confirmed that β-amyloid aggregates can generate the sustained activation of Akt, which affects the insulin response, tau phosphorylation (O’Neill et al., 2012), and the downstream effectors of the mTOR pathway that control cell growth, proliferation, and survival (Chong et al., 2010). Also, our data showed increased mTOR activity in the early and late stages of the 3xTg-AD model, suggesting that mTOR activity in the late phase may be closely related to high levels of hyperphosphorylated tau (Moreau et al., 2010; Caccamo et al., 2013) and that decreased levels of Bcl-2 may be related to cell death in the advanced stages of the disease (Rodriguez et al., 2011).

Conversely, we observed decreased levels of p-Akt Ser473, p-mTOR Ser2448, and mTOR activity concomitant with the decline of tauopathy and neuronal loss at 30 days post-ischemia in the absence of changes in Bcl-2 protein levels, suggesting apoptotic regulation (Iadecola and Anrather, 2011). Previous studies have reported that mTOR overactivation increases the cerebral infarct size (Zhang et al., 2012), and a decrease in mTOR activity reduces inflammation and nerve tissue damage and facilitates motor recovery after an ischemic event (Sekiguchi et al., 2012). In addition, Akt/mTOR inactivation promotes autophagy (Chong et al., 2012), and the inhibition of mTOR results in the degradation of hyperphosphorylated tau and NFTs through autophagy (Majumder et al., 2011; Jiang et al., 2014).

Autophagy failure has been widely associated with neurodegenerative diseases, which are characterized by the accumulation of proteins in an age- or injury-dependent manner (Madeo et al., 2009; Cuervo et al., 2010). Our findings suggest alterations on autophagy markers in the late stage of 3xTg-AD, resulting in increased levels of the Vps34 and Beclin-1 proteins at 6 months. While, the loss of this association was observed in the 3xTg-AD mice at 18 months. Furthermore, recent reports have demonstrated that these proteins are essential for macroautophagy initiation via autophagosome formation (Cebollero et al., 2012), and a loss of the association between these proteins significantly reduces macroautophagy (Kang et al., 2011; Jaber and Dou, 2012). In addition, the levels of LC3B-II, an autophagosome marker (Kabeya et al., 2000, 2004; Kuma et al., 2007), was increased in the early stage (6 months) in the 3xTgAD mice. However, a significant reduction was observed in the oldest mice (18 months), indicating an inverse relationship with tauopathy detected levels, which is supported by an inverse correlation between autophagy-related markers downregulation and the increase in tau aggregates (Meske et al., 2008; Majumder et al., 2011; Schaeffer et al., 2012; Ozcelik et al., 2013).

The upregulation of Beclin-1 levels after an ischemic event has been associated with the prevention of cell death by necrosis and a reduction in infarct volume (Carloni et al., 2008), being suggested Beclin-1 as a homeostatic regulator of autophagy post-ischemia (Carloni et al., 2010; Ryter et al., 2013). However, the specific role of Vps34 after acute injury remains unclear. Vps34 has been shown to contribute to exocytosis, autophagy, and the activation of mTOR (Backer, 2008). How Vps34 interacts with Beclin-1 to induce autophagosome formation is not clear yet. However, a recent report suggest that the location of Vps34 in specific cytoplasmic areas leads to autophagic vesicle formation (Burman and Ktistakis, 2010). This hypothesis is consistent with our observations of reduced levels of Vps34 total protein that were strongly associated with Beclin-1 at 30 days post-ischemia, which coincided with the decline of tauopathy levels in this time point. These data are consistent with the significant increase in LC3B puncta detection and its inverse relationship with hyperphosphorylated tau levels in the late phase post-ischemia. Nonetheless, an increase in LC3B-II on the first day post-ischemia has been reported in neuronal degeneration (Adhami et al., 2006; Li and McCullough, 2010), and it has been suggested that the pharmacological inhibition of autophagy reduces cerebral infarct volume (Kubota et al., 2010; Meloni et al., 2011; Gao et al., 2012). However, autophagic activity may play an important role in neuroprotection after an ischemic event, resulting in a beneficial effect on the cerebral cortex and hippocampus to prevent protein aggregation after stroke (Rami, 2009; Guo et al., 2010; Liu et al., 2010, 2013a,b; Zhou et al., 2011), and autophagic degradation has been proposed as an important regulator of hyperphosphorylated tau levels after an acute injury (e.g., in CI; Liu et al., 2010; Marino et al., 2011; Schaeffer and Goedert, 2012), which is consistent with our findings.

Moreover, CMA is activated under conditions of stress and is an interdependent process that cross-talks with macroautophagy via the upregulation of lysosomal proteins, such as Hsc70 and LAMP-2 (Gonzalez-Polo et al., 2005; Massey et al., 2006; Kaushik et al., 2008). Hsp90 is strongly involved in the hyperphosphorylation of tau in AD (Tortosa et al., 2009; Miyata et al., 2011; Salminen et al., 2011; Jinwal et al., 2013) and was increased in the 3xTg-AD mice at 6 months. However, a significant reduction in Hsc70 was observed in the older 3xTgAD mice (12 and 18 months). Hsc70 reduction has also been associated with the blockage of CMA (Cuervo et al., 1997; Kaushik et al., 2008) because Hsc70 is a substrate for degradation in the lysosome (Li et al., 2011; Kaushik and Cuervo, 2012). Therefore, our results showed that a number of proteins related to CMA, homeostasis, and cell survival were affected in the 3xTg-AD mice, which could enabling the aggregation of hyperphosphorylated tau.

Previous studies have demonstrated an increase in the expression of Hsc70, LAMP-2, and Hsp90 mRNAs, the proteins function together to remove protein aggregates and block neuronal apoptosis (Kobayashi et al., 2000) at 48 h after CI or cerebral hypoxia (Kitagawa et al., 1990; Kirino et al., 1991; Tanaka et al., 2002). These findings are consistent with our observations of increased Hsc70, Hsp90, and LAMP-2 levels and unchanged LC3BII levels in the intermediate stage post-ischemia (15 days). In contrast, in the late stage (30 days), there was an increase of macroautophagy-related marker, LC3BII, while there were not significant changes in the levels of Hsc70 and LAMP2 and a significant reduction in Hsp90 levels (Gonzalez-Polo et al., 2005; Massey et al., 2006; Kaushik et al., 2008).

Taken together, our findings maybe could support that the autophagic related markers indicate that this degradation pathway could be involved in reverses tauopathy, promotes cellular homeostasis, and plays a neuroprotective role via the inhibition of the mTOR pathway in late-stage of 2-VO CI model. These findings are in contrast to the decline of autophagic-related markers observed in 3xTg-AD mice model, which could enables tauopathy and neuronal loss at the late stage, suggesting together a differential temporal approach to the induction of neuroprotection and the prevention of neurodegeneration. However, additional experiments are needed for the understanding of the molecular mechanisms that regulate the degradation of protein aggregates by autophagy pathway according to the injury that alters cellular homeostasis.

## Author Contributions

JVO, design and acquisition data, analysis and interpretation data, manuscript preparation; GC-G design, analysis and interpretation data, manuscript preparation and critical revision. All authors read and approved the final manuscript.

## Conflict of Interest Statement

The authors declare that the research was conducted in the absence of any commercial or financial relationships that could be construed as a potential conflict of interest.
